# Models of mycorrhizal colonization patterns and strategies induced by biostimulator treatments in *Zea mays* roots

**DOI:** 10.3389/fpls.2022.1052066

**Published:** 2022-11-17

**Authors:** Victoria Pop-Moldovan, Larisa Corcoz, Valentina Stoian, Cristina Moldovan, Anca Pleșa, Sorin Vâtcă, Vlad Stoian, Roxana Vidican

**Affiliations:** ^1^ Department of Microbiology, Faculty of Agriculture, University of Agricultural Sciences and Veterinary Medicine Cluj-Napoca, Cluj-Napoca, Romania; ^2^ Department of Plant Physiology, Faculty of Agriculture, University of Agricultural Sciences and Veterinary Medicine Cluj-Napoca, Cluj-Napoca, Romania; ^3^ Department of Crop Plant, Faculty of Agriculture, University of Agricultural Sciences and Veterinary Medicine Cluj-Napoca, Cluj-Napoca, Romania; ^4^ Department of Grasslands and Forage Crops, Faculty of Agriculture, University of Agricultural Sciences and Veterinary Medicine Cluj-Napoca, Cluj-Napoca, Romania

**Keywords:** arbuscular mycorrhiza, arbuscules, vesicles, hyphal network, mycorrhizal maps, phenophase colonization

## Abstract

Agronomic inputs and technologies, especially fertilizers, act on the evolution of the symbiotic partnership between arbuscular mycorrhizal fungi and cultivated plants. The use of the MycoPatt method for the assessment of mycorrhizas in maize roots leads to the extraction of large parameter databases with an increased resolution over the colonization mechanism. The application of a biostimulator treatment on plants acted toward a reduction of root permissiveness for mycorrhizas. The phenomenon was noticeable through an increased colonization variability that overlapped with plant nutritional needs. The annual characteristic of the plant was highlighted by the simultaneous presence of arbuscules and vesicles, with a high share of arbuscules in the advanced phenophases. Colonized root parts presented numerous arbuscule-dominated areas in all phenophases, which indicated a continuous formation of these structures and an intense nutrient transfer between partners. Mycorrhizal maps showed the slowing effect of the biostimulators on colonization, with one phenophase delay in the case of biostimulated plants compared to the ones without biostimulators. The forecast models presented gradual colonization in plants without biostimulators, with the expansion of new hyphal networks. The use of biostimulators on plants exhibited a lower permissiveness for new colonization areas, and the mechanism relies on hyphae developed in the former phenophases.

## Introduction

Agriculture, in most developed countries, supports the economy, and the entire population depends on it (Aznar-Sánchez et al., 2019). It is a diversified sector; agricultural activities produce many jobs, and they contribute to the growth of the economy (Du Pisani, 2006). The world population, by 2050, will exceed 9.7 billion, and this will strain agricultural production due to increased demand for food (Verbruggen et al., 2013; Arora, 2019; Branco et al., 2021; Ganugi et al., 2022). The agricultural lands will be intensively processed due to agricultural management and the use of chemical fertilizers. These practices will lead to a destabilization of the agroecosystem (Carillo et al., 2020) resulting in soil and water pollution due to excess chemical fertilizers (Siedt et al., 2021). The microorganisms present in the soil are affected by these practices and also the entire ecosystem community (Gutiérrez-Núñez et al., 2022). According to studies presented by Maxwell et al. (2016), the intensification of agricultural activities endangers the biodiversity of the species present in the environment. To reduce the chemical inputs used (Xu and Geelen, 2018; Varia et al., 2022), a sustainable strategy has been adopted based on the application of biostimulators to crops, which make a significant contribution to increasing crop productivity (Xu and Geelen, 2018) and providing a positive impact on the environment (Mokgehle et al., 2021). The supply of organic food is of great interest to the global population. With the use of biological inputs, an increase in the quality of yields along with the maintenance of their quantity can be achieved (Boobalan and Nachimuthu, 2020; Kumar and Aloke, 2020). Corn, one of the world’s leading crops (Rehman et al., 2021), is a type of cereal that adapts to different environmental conditions and also different agro-climatic conditions (Reddy et al., 2021). Various fertilizers based on N, P, and K are used in this crop because maize is a plant with accelerated growth and needs a rich source of nutrients for production (Baghdadi et al., 2018).

Consequently, the use of chemical fertilizers represents a conventional practice, efficient for supplying plants with mineral nutrients, hence degrading the soil, the environment, and, at the same time, the entire agroecosystem (Halpern et al., 2015). In this practice, a beneficial alternative to maize cultivation is the use of biological inputs. These substances, also called biofertilizers, can be composed of different microorganisms that aim to increase plant productivity (Poveda et al., 2020). However, several plant biostimulators have been developed that act by facilitating the enzymatic and metabolic processes of plants, which are related to growth, development, and tolerance to various stresses (Xu and Geelen, 2018; Drobek et al., 2019). Rhizosphere microorganisms directly influence plant nutrition (Renaut et al., 2020). Subsequent research has shown that mycorrhizal fungi are a key group of microorganisms (Gao et al., 2020) that offer several benefits to crop plants. The improvement of nutrient absorption is the main advantage provided by these fungi, which can be visible in increased plant yields (Astiko et al., 2019). Arbuscular mycorrhizae represent a group of colonizing fungi that are extremely important in the rhizosphere (Xu et al., 2019). Present in the roots of the plants, they colonize several areas to a certain extent outside the roots, influencing both the plants and the other microorganisms in the soil (Nanjundappa et al., 2019). Mycorrhizae in agricultural ecosystems are the key mediators between hosts and soil conditions. Due to their specific role in adjusting the soil physicochemical conditions, these communities could improve soil fertility (Massenssini et al., 2015; Adamo et al., 2021). Throughout the analysis of fungal structures, it can be possible to highlight the plants’ needs (Johnson et al., 2012) and the plants’ efficiency in the use of inputs (Oehl et al., 2003), as well as the protection against harmful substances (Mukerji et al., 2012).

The main aim of the research was to assess the potential effect of a biostimulator application on mycorrhizal colonization in maize roots. The entire research analyzed the differences between the native mycorrhizal potential in the first phenophase and the colonization value in subsequent phenophases. Another aim was to test the performance of the MycoPatt method (Stoian et al., 2019; Stoian et al., 2022) to identify even the smallest differences produced by the biostimulator in the same fertilization conditions and to provide an increased resolution of colonization assessment. The secondary objectives follow a system of mycorrhizal assessment from simple to complex and were formulated as research questions: i) does the application of a biostimulator produce a quantifiable difference in average colonization parameters?; ii) is there a difference in the interdependence between colonization parameters related to the application of biostimulators?; iii) is the simultaneous presence of arbuscules and vesicles dependent on the combined effect of phenophase × treatment?; iv) is there a visible alteration of colonization strategies shared in the colonized roots after the application of biostimulators; v) do colonized roots exhibit different mycorrhizal patterns due to the applied treatment?; vi) is there a difference in the identification of the most important colonization parameters that can forecast the future development of colonization parameters in the roots?

## Materials and methods

### Field location and experimental design

The research studies were carried out in Iernut, Mureș County, Romania, in an experimental field established by the Microbiology laboratory, Faculty of Agriculture UASVM Cluj-Napoca. The field is located in the Transylvanian Plateau, N 46°27′13′ and E 24°14′0′. The soil type in the experiment was a phaeosiom, with N 0.159%, P 280 ppm, K 374 ppm, pH 7.62 and humus 2.94%. The biological material was represented by a Pioneer P9241 maize hybrid from the FAO 330 group. It is an early hybrid, showing a very good tolerance to different types of acid, degraded and low humus soils. It has a very well-developed root system and adapts very well to stressful factors (https://www.pioneer.com/).

The bifactorial research was carried out during the season of 2021. The base fertilization consisted in N 81 kg ha-1 and P 40.5 kg ha-1 (300 kg ha-1 commercial formula of NP 27-13.5), applied at sowing. In the 2-4 leaves growing stage, on half of the experiment field, it was applied the biostimulator AMER 6.3, which lead to the separation of plants in two categories: untreated ones (coded A1 in the experimental design) and the treated ones (coded A2 in the experimental design). Biostimulator composition consists in 6.6% total N (NH3-N 0.3%; organic N 6.3), 39.4 total Amino acids (13.0% free amino acids) and organic C 22%. The entire experiment followed the influence of biostimulator on mycorrhizal dynamics in roots in the most important growing stages from the vegetation period. Sowing date was 05 April 2021, with 72000 pl ha-1 density. The second factor (B) proposed in the experiment was the phenophase of plant growth. During the experiment, the most important moments for observing the mycorrhizal colonization were established as follow: B2 - phenophase of 6 leaves unfolded; B3 - phenophase of 8-10 leaves unfolded; B4- cob formation phenophase; B5- phenophase corresponding to physiological maturity (ripening). A control variant (coded A0) is added to the two factors, which presents the native mycorrhizal profile in 2-4 true leaves of the plants (B1) and is a comparison variant for the evolution of colonization during the entire vegetation period (coded A0-B1). Each plot had 250 m2, replicated 3 times.

### Laboratory and microscopic analyses

The research continued with microscopic analysis. Root samples after harvest from the experimental field were transported to the laboratory to begin microscopic analysis and to determine mycorrhizal fungal colonization parameters.

Maize roots went through four distinct steps in terms of the staining method before the microscopic preparation was made. The staining method was the one presented by (Vierheilig and Piché, 1998) but adapted by Stoian and Florian (2009) and supplemented by Stoian et al. (2022), which involves cleaning the roots with a 10% NaOH solution by immersion in this solution for 24 h. The next step was rinsing the roots with a water+vinegar solution for a few minutes. Root coloring involves preparing a solution of 5% ink, 5% white vinegar, and 90% distilled water and immersing the roots for 24 h. After this, the roots were rinsed with distilled water for the removal of an extra-staining agent. At the end of all the mentioned steps, the roots were prepared for microscopic analysis. Root segments (1-cm fragments) were placed on the slide, after which they were observed under a microscope. Thus, 15 root segments were analyzed in 15 microscopic fields for each experimental variant in three replications. The microscopic evaluation methodology was performed using the MycoPatt model (Stoian et al., 2019), which is an innovative model that shows the real position of the fungal structures present in the roots, calculates different indicators, and then generates automatically the mycorrhizal maps (Stoian et al., 2022).

### Data analysis

The microscopic assessment of all samples was conducted on a database of 6,075 observations, which served as a good basis for a detailed data analysis in R Studio software version 1.4.1106 (RStudio Team, 2019) under the R platform (RCore Team, 2021). The first step in data analysis was performed with package “psych” (Revelle, 2019), from which means, medians, and standard errors (s.e.) for each parameter were further used in the mycorrhizal assessment. ANOVA and least significant difference (LSD) tests, in the “agricolae” package (de Mendiburu, 2019), were used to evaluate the numerical differences between variants grouped by treatments and phenophases, with the “broom” package (Robinson et al., 2021) for the export of results. Pearson’s correlations between mycorrhizal parameters were used to evaluate the significance of interdependence between them, with formulas from the “Hmisc” package (Harrell et al., 2020). Arbuscules and vesicles from each treatment and phenophase were both analyzed in scatterplots, produced by the “stats” package from the R platform (RCore Team, 2021). Each set of data from treated and untreated variants was filtered and grouped based on intensity, arbuscules, and vesicles, in order to identify the number of clear colonization strategies (Corcoz et al., 2022b). Variants with an intensity lower than 10% were considered in the group of resistance colonization conditions; the intensity between 10% and 25% was assessed as proliferative colonization strategy; the roots with an intensity higher than 25% and an arbuscule/vesicle ratio higher than 1.0 were considered in the group of transfer colonization strategy; the roots with an intensity higher than 25% and an arbuscule/vesicle ratio lower than 1.0 were considered in the group of storage colonization strategy (Corcoz et al., 2022a). The strategy graph was performed with the package “scatterplot3d” (Ligges and Mächler, 2003). Two separate databases were created from the results obtained from untreated and treated plants, with each of them added to the observation from the A0-B1 assessment, which represents the native colonization profile. The two databases were analyzed through a principal component analysis (PCA) from the “vegan” package (Oksanen et al., 2019) and projected based on the phenophases and colonization strategies. This procedure resulted in four different PCA ordinations. The use of MycoPatt Excel for the analysis of microscopic observations produced 405 different mycorrhizal maps. The map database was filtered based on median values for each dataset associated with one treatment × phenophase combination; this procedure ensures the extraction of the most relevant mycorrhizal patterns. Additionally, the maps with the maximum arbuscules and vesicles from each treatment × phenophase combination were extracted to analyze the mycorrhizal expansion and developed structures in the roots. All maps were analyzed with an expanded version of the multi-point analysis method (Corcoz et al., 2022a), which permits the simultaneous comparison of different colonized areas from multiple maps. For each mycorrhizal parameter, starting with the second growth stage, the values of all parameters from the previous growth stages were used to create a forecast model. Each model was constructed step-by-step, with the Akaike information criterion (AIC) applied to all parameters, and they were grouped in a hierarchy based on their importance; the final model was analyzed for significance using the packages “caret” (Kuhn, 2020) and “MASS” (Venables and Ripley, 2002).

## Results

### Inter-phenophase differences induced by biostimulator application in colonization potential

Both frequency and intensity show high variations between growth stages for both treated and untreated plants (**Table 1**). The native colonization frequency exceeds 74% (A0-B1), a value that significantly decreases for both untreated and treated plants in the six-leaf growth stage. For the untreated plants, the colonization frequency increases over the control in the 8–10-leaf and cob formation stages, followed by a drastic reduction of a significant 15% at the end of the vegetation period. The treated plants show a significantly 10% higher reduction of frequency as compared to the untreated plants, a phenomenon amplified by the application of biostimulators. The intensity of colonization varied greatly between growth stages, with more than 40% at the beginning of plant growth, followed by a significant reduction in the six-leaf growth stage. Both the treated and untreated plants showed higher values of intensity in the 8–10-leaf and cob formation growth stages, followed by a significant reduction at full maturity. Arbuscules are the secondary structures with the highest ratio in colonized roots, but with a clear difference between the treated and untreated plants. While the first growth stage showed more than 12% of hyphae that formed arbuscules, the six-leaf stage showed a 7% reduction in the untreated plants and almost 10% in treated ones. Following the trend of intensity, the maximum arbuscule abundance was visible in the 8–10-leaf and cob formation stages, but with significantly lower values than at the beginning of growth and development. One interesting phenomenon was visible in the treated plants, where arbuscule abundance was set to 4%–5% from the 8–10 leaves up to the end of the vegetation period. Vesicles were restricted to less than 1% of colonized roots, with higher values in the last two growth stages for the untreated plants, compared to the cob formation stage for treated ones. These three values present significant differences compared to the native vesicular potential. Non-mycorrhizal areas varied in the interval of 53%–74%, with all plants presenting multiple uncolonized areas in the roots. All significant differences indicate a high permissiveness and root growth fluctuation, with higher values associated with growth stages that had reduced nutrient requirements.

**Table 1:** Differences between mycorrhizal parameters induced by the phenophase × treatment combined effect.

PxT	Frequency (%)	Intensity (%)	Arbuscules (%)	Vesicles (%)	Non-mycorrhizal areas (%)	Colonization degree (%)	Mycorrhizal/non-mycorrhizal area report	Arbuscule/vesicle ratio
A0_B1	74.75 ± 1.07ab	42.65 ± 0.86b	12.2 ± 0.60a	0.26 ± 0.06b	57.33 ± 0.86d	37.1 ± 0.98ab	1.29 ± 0.09a	0.26 ± 0.08bc
A1_B2	60.92 ± 1.09d	29.57 ± 0.61d	5.57 ± 0.33c	0.26 ± 0.05b	70.42 ± 0.61b	21.8 ± 0.71de	0.52 ± 0.01de	0.19 ± 0.06c
A1_B3	76.36 ± 0.92ab	46.13 ± 0.74a	9.43 ± 0.49b	0.23 ± 0.05b	53.86 ± 0.74e	39.0 ± 0.90a	1.34 ± 0.08a	0.42 ± 0.11abc
A1_B4	77.39 ± 0.94a	40.53 ± 0.65b	8.19 ± 0.39b	0.86 ± 0.09a	59.46 ± 0.65d	34.8 ± 0.78b	0.85 ± 0.02bc	0.84 ± 0.14a
A1_B5	62.73 ± 1.03d	30.09 ± 0.59d	3.00 ± 0.26de	0.76 ± 0.09a	69.90 ± 0.60b	22.4 ± 0.71d	0.53 ± 0.02de	0.38 ± 0.06bc
A2_B2	52.47 ± 1.18e	27.23 ± 0.73de	2.35 ± 0.24e	0.08 ± 0.02b	72.76 ± 0.73ab	19.4 ± 0.83de	0.56 ± 0.03de	0.18 ± 0.05c
A2_B3	71.74 ± 0.99bc	40.39 ± 0.79b	5.53 ± 0.31c	0.12 ± 0.03b	59.60 ± 0.79d	33.4 ± 0.93b	1.04 ± 0.04b	0.12 ± 0.05c
A2_B4	68.08 ± 1.10c	34.56 ± 0.72c	5.30 ± 0.36c	0.82 ± 0.11a	65.43 ± 0.72c	28.1 ± 0.84c	0.73 ± 0.03cd	0.67 ± 0.12ab
A2_B5	50.85 ± 1.17e	26.08 ± 0.70e	4.28 ± 0.36cd	0.24 ± 0.05b	73.90 ± 0.70a	18.2 ± 0.80e	0.50 ± 0.02e	0.44 ± 0.11abc
F test	88.47	104.53	66.78	19.48	104.53	91.72	44.65	6.04
*p*.val	<0.001	<0.001	<0.001	<0.001	<0.001	<0.001	<0.001	<0.001

Means ± s.e. followed by different letters present significant differences at p < 0.05 according to least significant difference (LSD) test.

A0-B1, control variant (native mycorrhizal profile) in phenophase 2–4 leaves; A1, untreated plants; A2, treated plants; B2, phenophase of 6 formed leaves; B3, phenophase of 8–10 formed leaves; B4, cob formation phenophase; B5, phenophase corresponding to physiological maturity.

The root volume colonized by mycorrhizae, expressed by the colonization degree, was set to 37% at the beginning of plant growth. Compared to this value, only the 8–10-leaf stage in the untreated plants showed a 2% increase. The six-leaf growth stage restricts the global mycorrhizal colonization value up to 19%–21% of the entire root system and extends this interval up to 18%–22% at the end of the vegetation period. A significant difference of more than 4% was visible between the untreated and treated plants, starting with the 8–10-leaf growth stage up to the full maturity of corn. Mycorrhizal/non-mycorrhizal area ratio is a synthetic parameter that follows closely the change in mycorrhizal share along with the roots. The beginning of the growth period (A0-B1) and the 8–10-leaf stage in the treated and untreated plants exhibit values higher than 1.0 of this parameter. This value represents an intense colonization process, with less uncolonized areas between colonized parts. At the end of the vegetation period, this ratio decreased significantly up to 0.5. Compared to the mycorrhizal/non-mycorrhizal area ratio, the ratio between arbuscules and vesicles varied less between the different growth stages. Even though the values of arbuscules far exceeded those of the recorded vesicles, the actual presence of one or both of these structures in the same colonized area produced variations in this ratio. The highest values were recorded in the cob formation stage, up to three times higher than in the previous stage for the treated plants and up to two times higher in untreated ones. At full maturity, the treated plants maintained higher values than untreated ones.

### Interdependence of mycorrhizal parameters related to the effect of biostimulator application

Pearson’s correlations were used to explore the connections between mycorrhizal parameters and their influence, which was related to their simultaneous presence in the same colonized root (**Table 2**). The native and untreated mycorrhizal profile shows a stable correlation coefficient (0.85) between the frequency and colonization intensity, a value with a small increase for the treated variants. Both untreated and treated profiles exhibit higher correlation coefficients for arbuscules and vesicles compared to the native profile. The differences were higher in terms of arbuscules determined by the presence of fungal components (frequency) and the development (intensity) in the roots. Regarding the vesicles, the ratio between arbuscule and vesicle correlation coefficients shows a five times higher value for arbuscules in native and untreated mycorrhizas, which is two times higher for treated variants. The colonization degree was more sensitive to the intensity observed in the roots, with an additional 0.09–0.13 value as compared to the frequency correlation coefficient. This parameter was highly correlated with the presence of arbuscules, indicating the need for extensive colonization prior to the development of arbuscules. In contrast, the correlation coefficients of colonization degree (0.14 = untreated variants and 0.09 = native variants) with vesicles indicate the possibility of appearance for these structures regardless of the dimension of the hyphal network. The treated variants show a different perspective; for the development of vesicles, at least a medium colonization is required to be developed. This sustains the presence of a storage mechanism only in deep colonized roots.

**Table 2:** Pearson’s correlation coefficients between mycorrhizal parameters under the combined effect of phenophase × treatment.

General	Intensity	Arbuscules	Vesicles	Non-mycorrhizal areas	Colonization degree	Mycorrhizal/non-mycorrhizal area report	Arbuscule/Vesicles ratio
Frequency	0.85	0.48	0.18	−0.85	0.89	0.46	0.15
Intensity		0.62	0.19	−1.00	0.99	0.68	0.19
Arbuscules			0.06	−0.62	0.61	0.48	0.25
Vesicles				−0.19	0.19	0.09	0.15
Non-mycorrhizal areas					−0.99	−0.68	−0.19
Colonization degree						0.67	0.19
Mycorrhizal/non-mycorrhizal area ratio							0.13
A0-B1	Intensity	Arbuscules	Vesicles	Non-mycorrhizal areas	Colonization degree	Mycorrhizal/non-mycorrhizal area ratio	Arbuscule/vesicle ratio
Frequency	0.84	0.44	0.09	−0.84	0.86	0.35	0.09
Intensity		0.55	0.10	−1.00	0.99	0.58	0.10
Arbuscules			−0.06^ns^	−0.55	0.54	0.34	0.15
Vesicles				−0.10	0.09	0.04^ns^	0.12
Non-mycorrhizal areas					−0.99	−0.58	−0.10
Colonization degree						0.57	0.10
Mycorrhizal/non-mycorrhizal area ratio							0.04^ns^
Untreated	Intensity	Arbuscules	Vesicles	Non-mycorrhizal areas	Colonization degree	Mycorrhizal/non-mycorrhizal area ratio	Arbuscule/vesicle ratio
Frequency	0.85	0.49	0.13	−0.85	0.88	0.48	0.11
Intensity		0.63	0.14	−1.00	0.98	0.70	0.15
Arbuscules			0.06	−0.63	0.62	0.51	0.21
Vesicles				−0.14	0.14	0.07	0.18
Non-mycorrhizal areas					−0.98	−0.70	−0.15
Colonization degree						0.70	0.15
Mycorrhizal/non-mycorrhizal area ratio							0.11
Treated	Intensity	Arbuscules	Vesicles	Non-mycorrhizal areas	Colonization degree	Mycorrhizal/non-mycorrhizal area ratio	Arbuscule/vesicle ratio
Frequency	0.87	0.50	0.24	−0.87	0.90	0.66	0.20
Intensity		0.64	0.30	−1.00	0.99	0.88	0.28
Arbuscules			0.13	−0.64	0.64	0.63	0.35
Vesicles				−0.30	0.29	0.26	0.13
Non-mycorrhizal areas					−0.99	−0.88	−0.28
Colonization degree						0.87	0.28
Mycorrhizal/non-mycorrhizal area ratio							0.33

Values marked with ns are considered not significant correlation coefficients at p < 0.05.

### Phenophase and treatment combined effect on the simultaneous presence of arbuscules and vesicles

**Figure 1:**
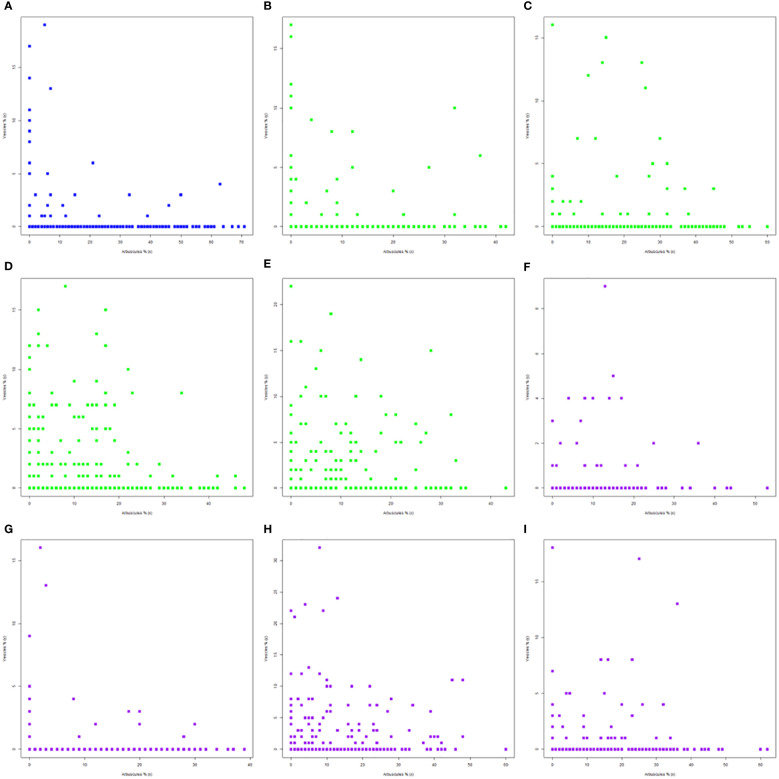
Simultaneous presence of arbuscules and vesicles in different phenophase under the influence of applied biostimulator: **(A)** A0-B1, **(B)** A1-B2, **(C)** A1-B3, **(D)** A1-B4, **(E)** A1-B5, **(F)** A2-B2, **(G)** A2-B3, **(H)** A2-B4, and **(I)** A2-B5. Legend: A0-B1, control variant (native mycorrhizal profile) in phenophase 2–4 leaves; A1, untreated plants; A2, treated plants; B2, phenophase of 6 formed leaves; B3, phenophase of 8–10 formed leaves; B4, cob formation phenophase; B5, phenophase corresponding to physiological maturity.

The simultaneous presence of arbuscules and vesicles can be analyzed by plotting them in the same scatterplot. The scatterplot approach permits a visual analysis of the data dispersion for each structure and the specific level where each structure was singular in colonized roots (**Figures 1A–I**). The native development of these structures was set to more than 70% for arbuscules and over 15% for vesicles (**Figure 1A**). The general visual position indicates the simultaneous presence of both structures at up to 10% of arbuscules and only 5% for vesicles. In addition to these cases, two different scenarios were possible for the native secondary structure development: the presence of arbuscules at up to 7%–8% along with 20% of vesicles, a scenario that implies less than 5% of cases, and the second scenario where 4%–5% of vesicles can be still present along with up to 60% of arbuscules. The absence of treatments in the A1-B2 stage led to the simultaneous presence of both structures of up to 8% for vesicles and 18% for arbuscules (**Figure 1B**). The difference of 10% was oriented toward an improved transfer instead of nutrient storage. Over these limits, vesicles could be present in up to almost 40% of arbuscules, without consistency. The roots in the next stage, A1-B3 (**Figure 1C**), showed the possibility of two simultaneous presence scenarios: the first one maintained the level of vesicles at 7%–8%, while the arbuscules could be present at up to 35%; the second one was oriented toward vesicle production of up to 15%, and the arbuscules were set to 27%. Both scenarios were present in stage A1-B4, with a larger dispersion of data (**Figure 1D**). Vesicle scenarios set these structures at up to 15% in the presence of up to 20% of arbuscules. The intense transfer scenario was maintained at up to 30% of arbuscules but decreased the level of vesicles by 5%. The final vegetation period, A1-B5 (**Figure 1E**), presented two distinct simultaneous presence scenarios: the storage scenario, with 20% of vesicles in the presence of only 10% of arbuscules, and the transfer scenario permits the presence of more than 30% of arbuscules with a drastic reduction of vesicles to 10%. The application of the biostimulator (**Figures 1F–I**) changed the simultaneous presence of both structures. The A2-B2 stage (**Figure 1F**) showed two possible arbuscules scenarios, both in restricted vesicle presence. The first one permitted only 4% of vesicles along with 20%–22% of arbuscules, while the increase of arbuscules in the interval of 22%–40% reduced the vesicles by up to 2%. Few observations indicated the possible presence of vesicles at up to 8%–9%, but only in the 10%–20% interval of arbuscules. The A2-B3 stage presented a change in the root colonization toward the formation of arbuscules and vesicles (**Figure 1G**). Both structures were present in higher values, along with the maintenance of previously identified scenarios. There were recorded multiple root parts where vesicles exceeded 15%, while for the arbuscules, their presence was more homogenous in the absence of vesicles. Starting with the A2-B4 stage, both structures increased their overall presence (**Figure 1H**). Vesicles grew to more than 30%, with 25% in a stable simultaneous presence with 15% of arbuscules. Arbuscules could have a 60% share of colonized roots and up to almost 50% of 10%–15% of vesicles. One interesting aspect is that at full maturity, A2-B5 (**Figure 1I**), the treated plants showed a drastic reduction to half of the previous values along with the maintenance of arbuscules. Similar to the trend visible in the A2-B2 stage, the roots with up to 22% of arbuscules were associated with 8%–9% of vesicles, while the increase of arbuscules up to 35% permitted the development of less than 5% of vesicles. Only some punctual colonization of the roots showed more than 15% of vesicles, but their presence was associated with both 0% and more than 30% of arbuscules, which makes them isolated cases.

### Colonization strategy profile due to the application of biostimulator

**Figure 2:**
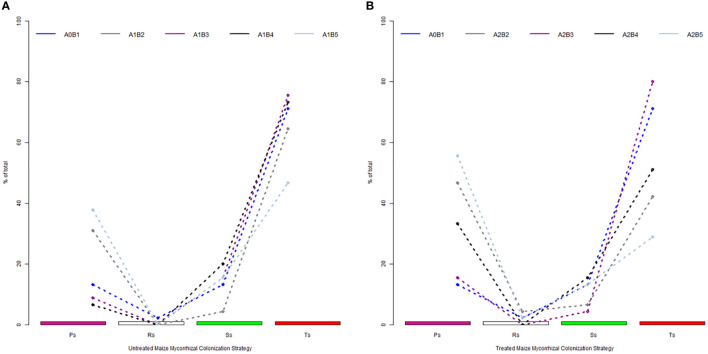
Specific phenophase colonization strategy shaped by the effect of biostimulator application: **(A)** untreated plants and **(B)** treated plants. Legend: A0-B1, control variant (native mycorrhizal profile) in phenophase 2–4 leaves; A1, untreated plants; A2, treated plants; B2, phenophase of 6 formed leaves; B3, phenophase of 8–10 formed leaves; B4, cob formation phenophase; B5, phenophase corresponding to physiological maturity; Ps, proliferation strategy; Rs, resistance conditions strategy; Ss, storage strategy; Ts, transfer strategy.

The analysis of mycorrhizal strategies based on observed colonization parameters revealed high differences between the native, incipient profile and further stage development of AM symbionts in the roots (**Figures 2A, B**). The native profile shows 2% of data in the resistance condition colonization. This value of resistance conditions was recorded at full maturity (A2-B5) and doubled (A2-B2) after the application of biofertilizers, both in the treated plants. Only 13% of colonized roots were oriented from the beginning toward the proliferation of hyphae, a mechanism that ensures a good hyphal network inside the roots for the future development of colonization. The next phenophases recorded a 2.5 times higher value (A1-B2) of proliferative strategy and almost three times higher at full maturity (A1-B5). An interesting aspect is that in the middle growth stages of the untreated plants (A1-B3 and A1-B4), the proliferative strategy shared from colonized roots was lower than 10%, being replaced by transfer or storage ones. For the treated plants, the proliferative strategy is variable, with a minimum recorded in A2-B3 and more than three to four times higher in the A2-B2 and A2-B5 growth stages. The presence of a transfer strategy, due to the overall abundance of arbuscules in colonized segments, was set to a maximum of 20% (A1-B4). Compared to this level, the treated plants in the same growth stage exhibited 5% fewer root segments oriented toward an intense transfer. The second growth stage (A1-B2 and A2-B2) was characterized by the poor presence of transfer strategies, a phenomenon that lasted even in the A2-B3 stage for the treated plants. In contrast, the native mycorrhizal colonization strategy profile was set up to almost 70% in the direction of storage. During the entire vegetation period of the untreated plants, the storage strategy exceeded 73% of colonized roots in the A1-B3 and A1-B4 growth stages and 80% of the treated plants in the A2-B3 stage. For the treated plants, the maximum storage strategy decreased by 30% in the A2-B4 and A2-B5 growth stages.

The use of the four colonization strategies has a high potential even when are applied on each analyzed root segment (**Supplementary Table 1**). By filtering the entire database, each of the segment show a clear orientation toward a colonization strategy. All microscopic fields from each position were counted for one of the proposed colonization strategies. Based on this approach, the variation intervals for each strategy can be accounted. Along the entire length of root segments in native mycorrhizal profile, the resistance strategy is visible in maximum 5 cases, while the average for proliferation is set to 10 cases. Storage strategy is similar to proliferative one in native profile, and the transfer one is present in more than half of the segment lengths. For untreated plants, as plant grow and reach each of the upper phenophases, the general strategy of root segments are oriented toward proliferation and transfer (A1-B2), respectively storage and transfer for (A1-B3 and A1-B4). Treated plants have a more equilibrated colonization strategy presence, with all colonization strategies – proliferative, storage and transfer – present in stages B2-B4 and a higher share for proliferative strategy in the final growth stage.

**Figure 3:**
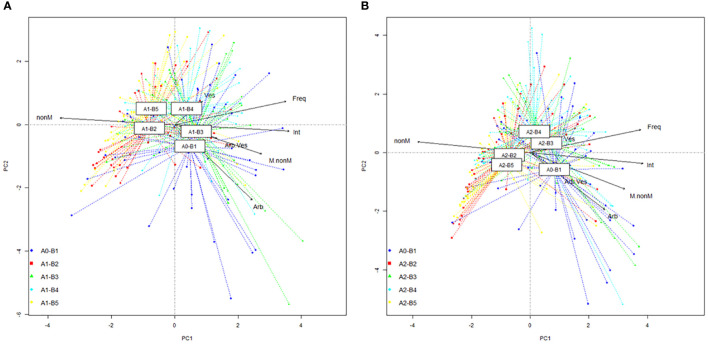
Principal component analysis (PCA) projection of phenophase-specific colonization: **(A)** untreated plants and **(B)** treated plants. Legend: A0-B1, control variant (native mycorrhizal profile) in phenophase 2–4 leaves; A1, untreated plants; A2, treated plants; B2, phenophase of 6 formed leaves; B3, phenophase of 8–10 formed leaves; B4, cob formation phenophase; B5, phenophase corresponding to physiological maturity; Freq, colonization frequency; Int, colonization intensity; nonM, non-mycorrhizal areas; M.nonM, mycorrhizal/non-mycorrhizal area ratio; Arb, arbuscules; Ves, vesicles; Arb.Ves, arbuscule/vesicle ratio.

PCA ordinations permit a good visualization of the entire colonization database, with the detection of centroids for each growth stage and the projection of vectors for each parameter (**Figures 3A, B**). For the untreated plants, the combined intensity–non-mycorrhizal area vector is folded almost perfectly on axis 1 (**Figure 3A**). The A1-B2 and A1-B5 centroids are along each part of the non-mycorrhizal vector, while A1-B3 and A1-B4 are positioned on each part of the Intensity vector. The second and final growth stages correlated with a decrease in colonization potential. Both datasets show a low dispersion on ordination, which is connected with a higher homogeneity of colonization. The position of A1-B3 and A1-B4, near the native colonization profile (A0-B1), indicates a high similarity in all recorded parameters. The A1-B4 stage position near frequency and vector is due to the highest values of both parameters within the entire database. The orientation of the combined vector (intensity–non-mycorrhizal areas) for the treated plants (**Figure 3B**), related to the native profile, is similar to the orientation observed in the untreated plants. Although the dataset of native colonization profile (A0-B1) maintains its position, the four colonization profiles associated with growth stages show a different positioning of centroids, a difference in data dispersion, and more importantly a different correlation with vectors. The second (A2-B2) and last (A2-B5) growth stages show the lowest data dispersion and the highest stability of colonization. Centroids of both growth stages have a nearby position, close to axis 1 of PCA. The growth stage A2-B2 is closer to the non-mycorrhizal vector, which indicates a higher susceptibility for plants in this growth stage to lose a share of the AM symbionts in their roots. An opposite image is observed by the analysis of the A2-B3 and A2-B4 datasets. The centroid of the third growth stage is located exactly on the vesicle vector and close to the frequency one. This position sustains the continuous development of vesicles along colonized roots at every moment and is correlated with the presence of hyphae. A slightly upper position of the A2-B4 growth stage centroid indicates colonization unrelated to the fast-continuous development of vesicles, but a continuous development of the previous fungal system developed in rots. Both native and A2-B3 centroids are positioned on both sides of the Intensity vector, which sustains the requirements of a clearly developed fungal network for the success of symbiosis functioning. An interesting aspect is the position of the A0-B1 centroid toward the arbuscule/vesicle ratio and arbuscule vectors. This position indicates the fast development of arbuscules in the roots of young plants, at a higher rate compared to vesicles, and the demand for higher nutrient transfer from AM fungi to plants. Also, the length of the roots reduced in this growth stage, which indicates, along with the position of the centroid near the mycorrhizal/non-mycorrhizal area ratio vector, two possible colonization scenarios. The first one is the development of a high fungal network, with a reduced formation of arbuscules and vesicles, which correspond to a proliferative strategy. The second one sustains a rapid formation of a higher share of arbuscules, instead of vesicles, concomitant with the development of hyphal networks. This scenario indicates an almost perfect symbiotic partnership that evolves simultaneously.

**Figure 4:**
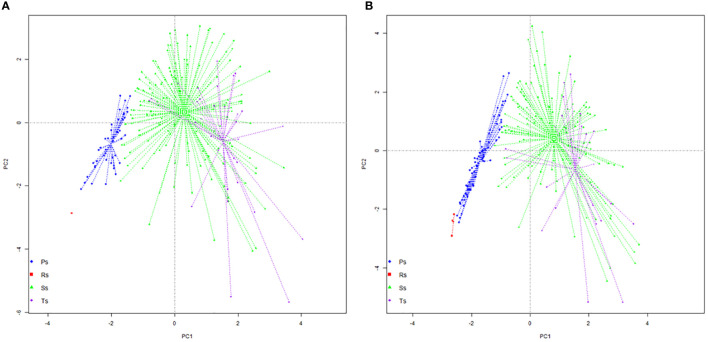
Principal component analysis (PCA) projection of specific colonization strategies: **(A)** untreated plants and **(B)** treated plants. Ps, proliferation strategy; Rs, resistance conditions strategy; Ss, storage strategy; Ts, transfer strategy.

Colonization strategy datasets of the untreated plants show a differentiated dispersion of data and the different locations of centroids in the four quadrants of PCA (**Figure 4A**). Only one resistance condition strategy is visible at a long distance from the proliferative strategy group. This indicates that the roots with a reduced permissiveness for AM symbionts will maintain these areas free of fungal structures. The proliferative group of data shows a transversal orientation with a reduced lateral dispersion. Most of the data recorded within this strategy group are similar, which can have multiple biological means. One hypothesis is that some root part has a lower permissiveness for symbionts, and their activity is reduced to a passing area for hyphae. Another hypothesis can be related to emergent colonization within newly formed roots, which can evolve in large hyphal networks within the next growth stages. Storage strategy exhibits the highest data dispersion, with a radial expansion towards the centroid. The center of the storage group is positioned near the middle of the ordination, which gives this group the highest importance in the entire ordination. The largest dispersion is observed within the group of transfer strategy data, with the position of the centroid close to the first third of axis 1. The length of lateral dispersion, along with very distanced points, indicates a heterogeneous arbuscule development within colonized roots. Resistance condition strategy in the treated plants is visible in a small group of data in the middle of the negative quadrat (−/−), with a reduced dispersion indicating the existence of very small differences between the roots with low permissiveness (**Figure 4B**). The proliferative strategy dataset was oriented transversally, with a very reduced lateral dispersion. The length between the lower and upper positioned points in this group indicates a difference in the dimension of the hyphal network between data. Also, it indicates that the same number of vesicles and arbuscules is developed in the roots encompassed in this group. Both storage and transfer strategy groups showed large dispersion of data, which indicates a heterogeneous development of each type of structure within the same type of colonized roots. This perspective indicates a potentially different function of each root segment within an entire root, with a primary orientation for the development of a fungal network. The dimension of the fungal network is the decision element for the secondary orientation of fungal strategy: either toward the production of vesicles and a clear storage strategy or toward the production of multiple arbuscules, which enhance the transfer from AM symbiont to plant.

### Specific phenophase mycorrhizal patterns as shaped by the biostimulator application

**Figure 5:**
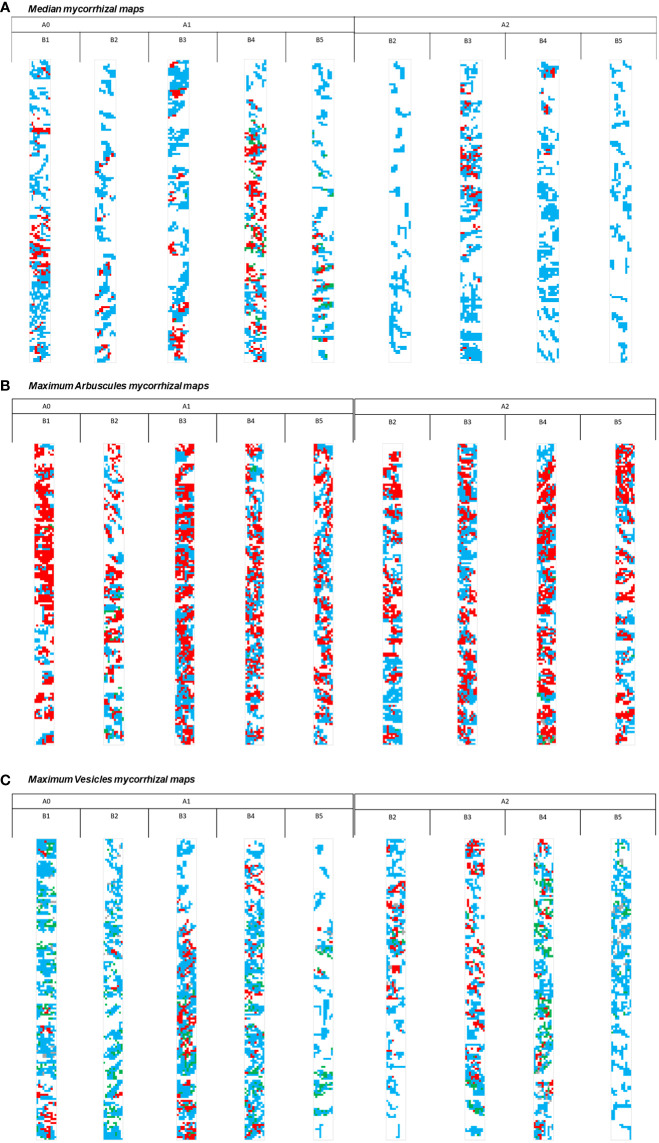
Mycorrhizal maps and colonization patterns shaped by the phenophase × treatment effect: **(A)** median colonization pattern, **(B)** maximum recorded arbuscules, and **(C)** maximum recorded vesicles. A0-B1, control variant (native mycorrhizal profile) in phenophase 2–4 leaves; A1, untreated plants; A2, treated plants; B2, phenophase of 6 formed leaves; B3, phenophase of 8–10 formed leaves; B4, cob formation phenophase; B5, phenophase corresponding to physiological maturity. Color coding in mycorrhizal maps: hyphae (blue), arbuscules (red), vesicles (green), and spores (gray).

The entire database consisted of 6,075 lines corresponding to 405 mycorrhizal maps and was further divided into 27 sub-databases. A large number of data implied a multi-step analysis for the extraction of the most relevant mycorrhizal maps for each growth stage × treatment combination. The Median for each combination was used to extract the maps corresponding to the middle of each sub-database. To this value, the Median− and Median+ were applied for the extraction of the upper and lower limits of mycorrhizal patterns, relevant to each sub-dataset. Additionally, two maps were extracted based on maximum values recorded for arbuscules and vesicles. Thus, the entire database was simplified to only 27 maps, relevant for all nine-leaf growth stage × treatment combinations. The resulting maps were further aggregated in three different comparative schemes (**Figures 5A–C**). Each scheme was analyzed based on the expanded multi-point analysis system, which permits the assessment of one map at a time of multiple points from different maps at the same time. The analysis of Median mycorrhizal maps (MMMs) revealed the longitudinal–lateral development of a dense hyphal network in newly formed plants (A0-B1 growth stage, **Figure 5A**). Arbuscules appeared to be grouped in 15%–20% of colonized roots, associated with complex hyphal networks. Only small areas lacked symbiont structures, with a reduced distance between the two colonized areas. In newly formed plants, the fungal symbiont extends gradually within colonized roots. The symbiont creates primary a hyphal sieve form in colonized roots, followed by the colonization of adjacent areas and further development of arbuscules. Compared to the development of fungal structures observed in the native mycorrhizal pattern (A0-B1), a similar one was observed in the treated plants, A2-B3 growth stage, with a reduced dimension and number of non-colonized areas. The native mycorrhizal profile shows a very dense arbuscule presence in the B4 growth stage consistent with an intense transfer between partners. This case, within an untreated plant group, is the maximum of fungal development. After the first growth stage (A0-B1), a decrease in the presence of fungal structures was observed. Hyphae developed rather laterally than longitudinally (A1-B2), which resulted in numerous, high-dimension, non-colonized areas, with the development of arbuscules in clusters. In addition to the fungal colonization strategy, in this growth stage, the roots developed faster than the possible fungal extension. The next growth stage (A1-B3) represented a reduction in root development along with an increase in fungal extension, with hyphae developing both longitudinally and vertically. Non-colonized areas appeared to be grouped, with an irregular form, and colonized areas were separated only by small free-mycorrhizal areas. Arbuscules occupied a high share in almost all mycorrhizal areas. The maximum colonization potential, with a dense and structurally diverse pattern, observed at the A1-B4 growth stage suffered a drastic decrease at the full maturity of plants. In the last growth stage, arbuscules appeared sporadically, along with vesicles, in mycorrhizal areas where the main extension was lateral. Non-mycorrhizal areas shared a similar pattern with the A1-B2 growth stage, alternating and separating colonized areas. The treated plants exhibited a far different mycorrhizal pattern than the untreated ones. A drastic reduction of fungal development was visible in the growth stage A2-B2, with large non-colonized areas and a lack of arbuscules. The hyphal development was oriented toward a longitudinal extension, with a reduced lateral development. The absence of arbuscules along with the sparse presence of colonized areas indicated the reset effect of the treatment toward the blocking of colonization. Between the A2-B2 and A2-B3 growth stages, a proliferation of fungal symbionts took place. Large hyphal networks and dense arbuscular areas were visible, with reduced non-colonized areas between the colonized ones. Both lateral and longitudinal extensions of the hyphae were visible, with the fungal component developing around a colonized point. The next growth stage (A2-B4) presented an inverse mycorrhizal pattern to the corresponding growth stage from the untreated group. While a lack of treatment stimulated the development of arbuscules, the application of biostimulators restricted drastically the development of arbuscules, leaving all the areas to be colonized by hyphae. In both cases, colonization was very present, but the potential transfer between the partners was lower in the treated plants. The comparison between the last stages in both groups of plants revealed an interesting phenomenon. For both mycorrhizal patterns, hyphae represented the support for the secondary development of arbuscules and vesicles. Even if the extension was reduced up to half of the roots, in the case of the untreated plants, secondary structures were present. In contrast, treatments restricted the development of secondary structures in the final development stage. Arbuscule abundance is a very good indicator of the intimate contact between the two partners and the enhanced transfer (**Figure 5B**). Based on mycorrhizal strategies, the analysis of the maximum observed arbuscularity enabled the forecast of potential intracellular expansion of AM hyphae. The native mycorrhizal potential (A0-B1) showed an intense presence of arbuscules, most of them grouped around hyphal networks. This image of arbuscule positioning indicated the formation of arbuscules concomitant with the extension of hyphae, but only in the most permissive part of the roots. The mycorrhizal map of native arbuscularity showed more than 50% presence of arbuscules, with sparse non-colonized areas and less than 20% of the root segments with discontinuities. Compared with this case, for the non-fertilized variants, a similar arbuscularity was reached once again in the B3 growth stage. This stage presented also a very good development of hyphae, reducing discontinuities to less than 5%. This map indicated the development of arbuscules in 50% of AM hyphae. The application of the biostimulator extended the duration, reaching a similar arbuscularity level with one growth stage (A1-B4). Arbuscules were visible in the entire root segment, with a starting point in the central area of the root. The overall image of the colonization trend revealed a difference in the expansion of fungal partners in the treated *vs.* untreated plants. For the untreated ones, the first growth stage was followed by a decrease in the dimension of hyphae and arbuscules (A1-B2). This mycorrhizal map presented a colonization base that sustained the proliferation of both arbuscules and hyphae in stage B3, which represented a peak in the arbuscule development. After this point, a slight decrease was visible in stage B4, with the presence of numerous uncolonized areas and an equal share of arbuscules and hyphae in the colonized space. The final growth stage made visible the presence of multiple uncolonized areas and a reduction of arbuscules of up to 30%–40% share of the total colonized root. For the treated plants, biostimulators acted as a slow-motion inducer for colonization. There was a visible decrease in colonization as compared to the native mycorrhizal profile, and also numerous uncolonized areas were visible. Arbuscules were not homogenous and distributed along the colonized roots, and the colonization direction was oriented for lateral expansion instead of a longitudinal one. The B3 stage revealed an increase in the dimension of the hyphal network with an arbuscularity similar to that of the previous growth stage. Numerous uncolonized areas were present, and the entire fungal development appeared separated. Compared to the untreated B3 stage, arbuscules were at half and hyphae at approximately 70%, which made this colonization more similar to the B4 stage in the untreated plants. The peak of arbuscules for the treated plants was reached in the B4 stage but showed only the development of arbuscules on a previously developed hyphal network. The final growth stage in the treated plants showed sparse colonization, with large uncolonized areas and a laterally oriented colonization. The share of arbuscules/hyphae was 40/60% in the total colonized areas.

An interesting aspect was that the maximum vesicles in new plants (A0-B1 stage) were equal to the arbuscules developed (**Figure 5C**). The large hyphal network that sustained the development of vesicles presented disruption points, with large uncolonized areas. Starting with the B2 stage, in the untreated plants, there was visible a decrease in hyphal expansion in the areas where vesicles were present. This stage presented a reduction point in the overall colonization, followed by an increase in hyphae and arbuscules (A1-B3 stage), the maintenance of the hyphal network in the B4 stage, and a sudden decrease in the final growth stage. The untreated plants showed a reduced homogeneity of mycorrhizal colonization in the areas where vesicles had the highest abundance. The final growth stage presented very large uncolonized areas, a chaotic orientation of colonization, and the presence of only a few vesicles. In contrast, the intense period of growing in the treated plants (A2-B2/B3 and B4 stages) was marked by the presence of numerous arbuscules, a homogenous colonization with small uncolonized areas, and an increased presence of vesicles from one stage to another. Interestingly, the final growth stage in the treated plants (A2-B5) showed a large colonized area, with vesicles still present, but no arbuscules.

### Forecast models of colonization parameters due to the biostimulator effect

The large database resulting from the analysis of mycorrhizas in the roots of maize permitted further exploration of their assemblage in forecasting the colonization parameters in each of the subsequent growth stages. For the forecast of colonization in the B2 growth stages, the parameters from the native profile were used. The difference between the two obtained models was induced by the application of biostimulators in the B0 growth stage (**Supplementary Tables 2, 3**). The frequency model showed the first visible difference between the treated and untreated plants, due to the Intercept value. The untreated plants started with a base value of 60.28% frequency, to which was added 2.4% for each percent of intensity. For the treated plants, the model for frequency in the B2 stage relied on multiple parameters. Each percent of vesicles was multiplied by 1.86% in the frequency, to which was added 0.09% from the value of colonization degree. Arbuscules decreased slightly the frequency with 0.17% for each 1% of their presence. Intensity presented a similar model for the two types of colonization. The untreated plants started from 29%, to which was added almost 1% for each percent of vesicles developed by fungi. The treated plants exhibited an intensity of 25%, to which was added more than 1.64% from the vesicles’ presence and 0.05% from colonization degree, but the arbuscules decreased the final value at 0.11% for each 1% of their presence. An interesting case was the forecast of arbuscules in the untreated plants, which started from a base value of 3.36%, to which was added almost 0.1% for each 1% of frequency. This value was decreased by the observed value of arbuscules in the initial growth stage. Based on the AIC model, the arbuscules in the untreated plants had a minimum value of 3.36% in the second growth stage, a value to which was added almost 0.1% for each 1% of the observed frequency, and a slight decrease due to the level of arbuscularity. The vesicles in these plants were slightly dependent on the intensity of colonization, with these structures designed for storage not being obligatory for the colonization mechanism in this stage. For the treated plants, the model sustained the development of arbuscules in close relation with vesicles developed in the previous stage. The high value of intercept was annulated by the negative score given to both intensity and the presence of uncolonized areas, which both sum 100%. The final model presented the importance of vesicles similar to the one in the frequency and a positive influence of the mycorrhizal/non-mycorrhizal area ratio. The forecast of colonization in the final growth stage presented highly different models that forecast the potential level of mycorrhizal parameters. The untreated plants started from almost 89% of the expected frequency, to which was added 1.12% from vesicles observed in the first and cob formation growth stages, with 6.35% applied to the value of the mycorrhizal/non-mycorrhizal area ratio. The final value was decreased by the colonization degree in the previous growth stage (more than 0.4/each 1% of colonization degree), with the mycorrhizal/non-mycorrhizal area ratio at 1.16 from the first growth stage and the arbuscules from the second growth stage. The treated plants had a different forecast of frequency, with a starting value of only 36.1%. This value contributed to the vesicles observed in the first and fourth growth stages (2.12/0.13) and the arbuscule/vesicle ratio in the second and fourth growth stages (0.54/0.2). The decreases in the model represented the values of intensity in the second growth stage, the mycorrhizal/non-mycorrhizal area ratio, and non-mycorrhizal areas in the third stage, with the frequency observed in the fourth stage. The difference in the base of modeled intensity, between the untreated and treated plants, was more than 15%. The untreated plants had a final colonization intensity that started from 44.4%, to which was added the frequency (0.09) and vesicles (0.59) in the first growth stage, with the vesicles (0.64) and the mycorrhizal/non-mycorrhizal area ratio (4.61) in the fourth growth stage. The decrease in the final value was due to the first colonization degree, the arbuscules and the dimension of uncolonized areas in the second growth stage, the colonization degree in the third growth stage, and intensity in the fourth growth stage. For the treated plants, the model increases were based on vesicles in the first and fourth growth stages, with the arbuscules and arbuscule/vesicle ratio in the third growth stage. An advantage of the final value was due to the mycorrhizal/non-mycorrhizal area ratio. The intensity decreased due to the frequency at the beginning of growth, the intensity in the second growth stage, the dimension of uncolonized areas in the third growth stage, and the colonization degree in the fourth growth stage. Arbuscules had high expectation presence even at the end of the vegetation period in the untreated plants. They started at 3.04%, which contributed significantly to the mycorrhizal/non-mycorrhizal area ratio from the fourth growth stage and with a score of 1.59. In contrast, for the treated plants, the intercept value for the model was negative, and a high contribution was due to the colonization degree in the second growth stage (1.56). Vesicles were expected to have a reduced presence in both the untreated and treated plants at the end of the vegetation period, with both models presenting a potential presence of approximately 1% maximum.

## Discussions

### Factors that intervene in the fungi–host symbiotic process

Climate, soil nutrients, soil, and the stage of growth of the host plant are all significant factors in mycorrhizal colonization. Mycorrhizae represent a microbial community very sensitive to abiotic factors. Thus, the evaluation of this community provides valuable information about the footprint left by different agricultural techniques and the type of fertilization. Soil is a decisive factor in mycorrhizal colonization. Fungal colonization parameters are negatively correlated with the number of spores and with the pH level (Govindan et al., 2020), and the level of humidity positively influences the development of fungal colonization (Oliveira and Oliveira, 2010). Thus, mycorrhizae show extensive growth in moist soils. Mycorrhizae adapt to different types of soil; Clark and Zeto (1996) showed significant differences in mycorrhizal colonization of maize roots in acidic and alkaline soils. Aguegue et al. (2017) demonstrated the effectiveness of mycorrhizal fungi to promote good plant development in soil with a pH of 5.1. Other authors have the opinion that a fungistatic effect can be present for mycorrhizal spores in acid soil, as a germinative substrate in maize culture, and can modify mycorrhizal development by suppressing it (Siqueira et al., 1984). The diameter of the root is very important for the colonization of the roots by arbuscular mycorrhizas. de Vries et al. (2021) showed that colonization is positively correlated with root diameter but negatively correlated with its length. Plants with a large root diameter, but few in number, are dependent on mycorrhizal fungi colonization, while plants with thin and multiple roots are not dependent on mycorrhizae. Because of the thin dimension of these roots, they can acquire the necessary nutrients without the need for mycorrhizal symbiosis (Zou et al., 2019). The period from two to four leaves to six leaves is characterized by a high increase in the root system development (Liu et al., 2019), at a speed that exceeds the hyphal potential extension and leads to a reduction in the colonization parameters. During the growth stage of a plant known as the flowering phenophase, Bender et al. (2019) found an increase in the number of fungal hyphae and vesicle-like structures. The untreated plants show a colonization fluctuation, due to the intensity of plant growth, in growth stages characterized by high nutritional requirements for mycorrhizas being highly present in the roots (Sheteiwy et al., 2021). The application of biostimulators increases root growth and decreases the permissiveness for the mycorrhizal partner, up to 50% for frequency and 26%–27% for intensity. The annual characteristic of the plant is very visible in the development of secondary structures, with a higher ratio of arbuscules and less than 1% of vesicles present in the roots. This phenomenon implies high-speed nutrient exchanges between arbuscules and root cells, associated with the plant growth needs and only a small part of nutrients deposited in vesicles (Whiteside et al., 2019). Once the treated plants reach the 8–10-leaf phenophase, a climax of colonization is visible in the arbuscule abundance. The intensity of transfer through arbuscules remains balanced, with no significant changes in their presence in the roots up to the end of the vegetation period. This phenomenon implies the stability of root growth and permissiveness, which is established by the application of biostimulators, which maintain the arbuscule development potential at high values (Garg and Bharti, 2018). Another factor with an important role in the process of fungal colonization is given by the inputs brought into the agricultural ecosystem. Aguegue et al. (2017) stated that the frequency and intensity of mycorrhizal colonization are strongly influenced by the application of N–P–K-based treatments. Several authors believe that a variety of mycorrhizal species are found in soils with a minimal tillage system and low fertilization (N and P) in the case of maize cultivation (Baltruschat et al., 2019). Phosphorus is an element that maize needs to have efficient growth and considerable yield. However, many studies have shown that a high concentration of phosphorus inhibits the development of mycorrhiza; also in the study presented by Sheng et al. (2012), phosphorus reduced mycorrhizal colonization but increased root density.

### Fungal strategies in maize roots

The application of threshold values for colonization strategies eliminates the masked trends from the entire database. As a general overview, the higher average values of arbuscules indicate a good connection between the two partners, and less than 1% of vesicles in each variant have a reduced necessity for storage. In the strict sense of colonization strategies, the threshold of 25% intensity of colonization in conjunction with an arbuscule/vesicle ratio lower than 1.0 indicates a clear storage strategy, while a ratio higher than 1.0 indicates a clear transfer strategy. The use of colonization strategies permits the separation of colonized roots into segments with the differential oriented mechanism. For both the treated and untreated plants, the reduced number of root segments oriented clearly toward a transfer strategy is associated with a constant flow of nutrients transferred to plants by the fungal partner. This is based on the continuous development of the roots, with the formation of approximately the same number of arbuscules continuously, while the old ones are digested by the plant. This creates active arbuscules spots alternating with inactive/digested arbuscules spots in the roots. Arbuscules as mycorrhizal structures have a short life span and disappear after a few days of formation, continued by the formation of new arbuscules (Montero et al., 2019). Vesicles, however, are long-life storage structures, with a primary role in nutrient storage, a deposit that is released during the plant growth stages with higher nutritional requirements (Ortiz-Ceballos et al., 2007; Meyer et al., 2021). Even the low general values of these structures are present along the entire root and remain present after the arbuscules are digested. This indicates the presence of older colonized areas, where the hyphal network plays a distribution hub role and is secondary as a storage area. Continuous formation of new vesicles indicates an overall much greater potential of AM symbionts to absorb nutrients than the rate of plant consumption (Smith and Smith, 2011). According to Bueno de Mesquita et al. (2018), mycorrhizal colonization can influence some plant species’ biomass and plant growth time (phenophase). The map of the B4 stage in the treated plants reveals a stage-by-stage colonization, with the development of hyphae prior to the intracellular development of arbuscules. The process indicates the need for an extracellular contact between fungi and root cells, with an exchange of recognition signals, which will act as a key for cell permissiveness in the acceptance of arbuscule development. In contrast, the absence of the biostimulator sustains the gradual colonization of plant roots, which is visible in both hyphal and arbuscule development in the same growth stage. Vesicle development in the roots of maize is a perfect indicator of colonization potential. Due to the annual characteristic of this species, the need for storage structures is reduced, and vesicle presence can be correlated with lower growth intensity of plants and also with high availability of nutrients. The root segments where vesicles are formed present lateral hyphae branching and the association with arbuscules. This indicates the absence of root parts where storage is the main role. The variations observed in all mycorrhizal maps reveal a transition characteristic of vesicles, with short-time storage potential in case of increased nutrient absorption from the soil by AM fungi (Varga et al., 2015). The absence of arbuscules in areas where vesicles are present, at the end of the vegetation period of the treated plants, indicates the presence of storage specialized root areas. Combined with the necessity of fungi to assure a reserve of nutrients for future development after a plant is removed from the field, this phenomenon shows the area from the roots that will act as nutrient support. Bender et al. (2019) observed an intensification of the increase in the number of fungal hypha- and vesicle-like structures in the flowering phenophase of a plant.

### Heterogeneity of mycorrhizal development in different phenophases

For both frequency and intensity of colonization in the treated plants, in the second growth stage, the development of vesicles in the first growth stage represents the main parameter for increase. This phenomenon indicates the potential role of vesicles in the short-term storage of nutrients necessary for the construction of new hyphae (Cui and Caldwell, 1997). In contrast, the absence of the biostimulator in the untreated plants forces the maintenance of the colonization in a gradual form, relying on the hyphal network that developed inside the colonized roots to colonize new areas. For the extension of hyphae, which is the intensity of fungal presence, the vesicles are the most important parameter, which indicates the same short-storage role. The untreated plants produce constant arbuscules, in newly colonized areas, a mechanism that implies an intimate contact between the two partners and a permanent evolution of the symbiosis. In these plants, vesicles are not expected to be formed, due to the increased need for plants for nutrients. Arbuscules in the treated plants are highly dependent on the vesicles formed in the roots colonized in the early stage of plant development. Similar to the phenomenon observed for hyphae, these plant vesicles play an important role in the construction of penetrant hyphae that form arbuscules inside the root cells. In contrast, vesicles are not expected in the second growth stage, when the hyphal network is extended in the roots and the need for nutrients requires arbuscules for an increased transfer. At the end of the vegetation period, the untreated plants present high colonization, with a dense hyphal network and arbuscules present in cells. The entire growing season implies constant colonization of newly formed (or extended) roots. Vesicles present in the cob formation stage sustain the development of new hyphae and the colonization of new areas in the roots. Based on the parameters included in the forecast model, frequency implies multiple points of colonization but with a reduced lateral or longitudinal branching which will be visible in the roots in alternating colonized with uncolonized areas. The treated plants have a reduced permissiveness for the development of new colonization points, with the potential mechanism relying on the hyphal network already present in the roots, which are extended inside the roots along with their growth. Vesicles are visible in the intensity models of both the untreated and treated plants. Due to their presence and the dispersion in colonized roots, they play a hub role in the development of lateral and longitudinal hyphae, which creates the network that defines the actual intensity. The observed values of these structures were reduced, which can indicate an ephemeral characteristic of vesicles and a potential regulator role of the nutrient flow in the hyphal network. Arbuscules maintain their presence until the end of the vegetation period in untreated plants. This phenomenon is due to the need of plants for symbionts in the improved acquisition of nutrients and a higher transfer inside root cells. An intensity of over 50% in the fourth growth stage can lead to an increase of arbuscules at up to 5% in the final one. The treated plants exhibit a different model of arbuscule presence, with an important role in the entire hyphal extension from the second growth stage. Based on this value and the multiplication potential, arbuscules can reach an equal share in colonized roots with hyphae. In both cases, at the end of the vegetation period, vesicles have a reduced presence, which is consistent with the formation of these structures only for fungal needs and not for plants. The efficiency of the symbiotic process, in addition to those listed above, can also be governed by temperature (Kilpeläinen et al., 2020), the presence of heavy metals (de los Angeles Beltrán-Nambo et al., 2021), and the diversity of fungal species (Jansa et al., 2008).

## Conclusion

The native mycorrhizal potential of maize is set to 74% frequency and more than 40% intensity of colonization, with a slight decrease in the untreated plants and a higher reduction for the treated plants until the end of the vegetation period. All plants showed multiple uncolonized areas, with unequal lateral and longitudinal development of hypha during the entire life cycle of plants. Arbuscules and vesicles have a native simultaneous presence of 10% and 5%, respectively; with these values, the root segment has a clear orientation for the development of only one of the structures. The untreated plants have a reduced potential for developing colonization in resistance conditions, while for the treated ones, this colonization mechanism is visible immediately after the application of the biostimulator. Mycorrhizal maps show a continuous development of arbuscules associated with a constant flow of nutrients from the fungal partner toward the plant, independent of the applied treatment. The overall colonization pattern, for both the treated and untreated plants, indicates a reduced presence of large uncolonized areas; in most parts of the roots, these areas are dispersed and small in size. In the entire root system, uncolonized discontinuities tend to be colonized in advanced phenophases, with the development of large hyphal networks, or a combination of hyphae and arbuscules.

## Data availability statement

The original contributions presented in the study are included in the article/**Supplementary Material**. Further inquiries can be directed to the corresponding author.

## Author contributions

V-PM and RV conceived and designed the research. V-PM, LC, VlS, and RV performed the experiments. LC and VlS analyzed the data. VaS, CM, AP, and SV analyzed the literature related to phenophase and crop technologies. All authors prepared and wrote the first draft and the final form of the manuscript. All authors contributed to this article and approved the submitted version.

## Acknowledgments

This article is part of a PhD study on the thematic area of “*Zea mays* mycorrhizal patterns driven by agronomic inputs” conducted by the first author V-PM, under the coordination of RV.

## Conflict of interest

The authors declare that the research was conducted in the absence of any commercial or financial relationships that could be construed as a potential conflict of interest.

## Publisher’s note

All claims expressed in this article are solely those of the authors and do not necessarily represent those of their affiliated organizations, or those of the publisher, the editors and the reviewers. Any product that may be evaluated in this article, or claim that may be made by its manufacturer, is not guaranteed or endorsed by the publisher.
