# Supplementary material for: Models of mycorrhizal colonization patterns and strategies induced by biostimulator treatments in Zea mays roots
Source: Front Plant Sci. 2022 Nov 17;13:1052066. doi: 10.3389/fpls.2022.1052066 (PMC9713310; doi:10.3389/fpls.2022.1052066)
Supplement: Supplementary file 1 [file DataSheet_1.docx]

Supplementary Material

# Supplementary Tables

Supplementary Table 1. Synthesis of segment-colonization strategy orientation due to the interaction phenophase *x* treatment

|  | A0-B1 | | | | A1-B2 | | | | A1-B3 | | | | A1-B4 | | | | A1-B5 | | | | A2-B2 | | | | A2-B3 | | | | A2-B4 | | | | A2-B5 | | | |
| --- | --- | --- | --- | --- | --- | --- | --- | --- | --- | --- | --- | --- | --- | --- | --- | --- | --- | --- | --- | --- | --- | --- | --- | --- | --- | --- | --- | --- | --- | --- | --- | --- | --- | --- | --- | --- |
| MF | Rs | Ps | Ss | Ts | Rs | Ps | Ss | Ts | Rs | Ps | Ss | Ts | Rs | Ps | Ss | Ts | Rs | Ps | Ss | Ts | Rs | Ps | Ss | Ts | Rs | Ps | Ss | Ts | Rs | Ps | Ss | Ts | Rs | Ps | Ss | Ts |
| 1 | 4 | 11 | 8 | 22 | 4 | 18 | 8 | 15 | 0 | 9 | 16 | 20 | 0 | 12 | 10 | 23 | 0 | 19 | 17 | 9 | 3 | 27 | 5 | 10 | 0 | 15 | 12 | 18 | 1 | 13 | 13 | 18 | 4 | 24 | 7 | 10 |
| 2 | 5 | 8 | 6 | 26 | 3 | 15 | 8 | 19 | 0 | 10 | 11 | 24 | 1 | 11 | 10 | 23 | 0 | 20 | 11 | 14 | 3 | 25 | 6 | 11 | 3 | 14 | 10 | 18 | 3 | 15 | 9 | 18 | 8 | 18 | 6 | 13 |
| 3 | 4 | 6 | 12 | 23 | 4 | 14 | 8 | 19 | 0 | 4 | 15 | 26 | 2 | 7 | 10 | 26 | 1 | 20 | 15 | 9 | 6 | 24 | 5 | 10 | 1 | 12 | 11 | 21 | 0 | 16 | 11 | 18 | 5 | 22 | 4 | 14 |
| 4 | 1 | 6 | 9 | 29 | 7 | 15 | 5 | 18 | 1 | 5 | 14 | 25 | 1 | 8 | 10 | 26 | 3 | 17 | 14 | 11 | 8 | 17 | 8 | 12 | 1 | 12 | 9 | 23 | 2 | 13 | 11 | 19 | 7 | 21 | 6 | 11 |
| 5 | 4 | 7 | 7 | 27 | 2 | 20 | 7 | 16 | 0 | 7 | 15 | 23 | 1 | 5 | 13 | 26 | 2 | 21 | 9 | 13 | 5 | 25 | 4 | 11 | 1 | 12 | 11 | 21 | 5 | 12 | 9 | 19 | 10 | 19 | 6 | 10 |
| 6 | 5 | 8 | 10 | 22 | 3 | 20 | 6 | 16 | 0 | 8 | 11 | 26 | 1 | 8 | 10 | 26 | 2 | 16 | 16 | 11 | 8 | 19 | 10 | 8 | 0 | 14 | 11 | 20 | 1 | 15 | 9 | 20 | 7 | 21 | 6 | 11 |
| 7 | 4 | 9 | 8 | 24 | 1 | 23 | 5 | 16 | 0 | 8 | 12 | 25 | 1 | 5 | 13 | 26 | 1 | 20 | 12 | 12 | 4 | 23 | 10 | 8 | 0 | 12 | 13 | 20 | 4 | 11 | 10 | 20 | 10 | 20 | 8 | 7 |
| 8 | 4 | 7 | 8 | 26 | 4 | 16 | 8 | 17 | 0 | 5 | 16 | 24 | 0 | 7 | 14 | 24 | 3 | 17 | 17 | 8 | 7 | 22 | 7 | 9 | 0 | 12 | 17 | 16 | 2 | 17 | 10 | 16 | 7 | 22 | 8 | 8 |
| 9 | 2 | 8 | 6 | 29 | 4 | 19 | 9 | 13 | 0 | 7 | 16 | 22 | 1 | 7 | 10 | 27 | 2 | 12 | 22 | 9 | 3 | 25 | 12 | 5 | 0 | 15 | 10 | 20 | 2 | 14 | 12 | 17 | 6 | 23 | 7 | 9 |
| 10 | 3 | 6 | 7 | 29 | 7 | 12 | 7 | 19 | 0 | 2 | 16 | 27 | 2 | 10 | 8 | 25 | 3 | 17 | 15 | 10 | 5 | 22 | 8 | 10 | 0 | 13 | 12 | 20 | 4 | 11 | 12 | 18 | 5 | 22 | 8 | 10 |
| 11 | 3 | 9 | 6 | 27 | 5 | 16 | 5 | 19 | 0 | 5 | 20 | 20 | 1 | 10 | 14 | 20 | 5 | 21 | 11 | 8 | 7 | 18 | 12 | 8 | 1 | 14 | 12 | 18 | 3 | 15 | 10 | 17 | 6 | 23 | 5 | 11 |
| 12 | 4 | 4 | 7 | 30 | 2 | 17 | 9 | 17 | 0 | 5 | 22 | 18 | 0 | 13 | 10 | 22 | 2 | 19 | 14 | 10 | 6 | 23 | 8 | 8 | 1 | 11 | 15 | 18 | 5 | 17 | 11 | 12 | 7 | 23 | 7 | 8 |
| 13 | 4 | 10 | 6 | 25 | 4 | 20 | 4 | 17 | 2 | 11 | 11 | 21 | 1 | 12 | 10 | 22 | 2 | 18 | 16 | 9 | 10 | 17 | 9 | 9 | 0 | 13 | 15 | 17 | 1 | 17 | 10 | 17 | 6 | 27 | 2 | 10 |
| 14 | 3 | 11 | 5 | 26 | 2 | 20 | 8 | 15 | 0 | 8 | 15 | 22 | 0 | 10 | 11 | 24 | 4 | 17 | 11 | 13 | 3 | 24 | 10 | 8 | 2 | 10 | 16 | 17 | 2 | 15 | 11 | 17 | 6 | 20 | 9 | 10 |
| 15 | 2 | 13 | 4 | 26 | 0 | 23 | 7 | 15 | 1 | 9 | 12 | 23 | 1 | 9 | 13 | 22 | 3 | 16 | 17 | 9 | 4 | 24 | 9 | 8 | 0 | 12 | 13 | 20 | 1 | 19 | 13 | 12 | 10 | 21 | 6 | 8 |

Supplementary Table 2. Stepwise regression models for forecasting the colonization parameters in untreated plants

|  | F2 | I2 | A2 | V2 | F3 | I3 | A3 | V3 | F4 | I4 | A4 | V4 | F5 | I5 | A5 | V5 |
| --- | --- | --- | --- | --- | --- | --- | --- | --- | --- | --- | --- | --- | --- | --- | --- | --- |
| Intercept | 60.28 | 29.31 | 3.36 | -0.01 | 65 | 37.86 | 829.27 | 2.99 | 86.3 | 37.29 | 8.99 | 3.73 | 88.96 | 44.4 | 3.04 | -0.780 |
| F1 |  |  | 0.09 |  | 0.14 | 0.08 | 0.06 |  |  |  | 0.02 | -0.008 |  | 0.09 | 0.04 | 0.01 |
| I1 | 2.4 |  |  | 0.006 |  |  | -8.03 |  |  |  |  |  |  |  |  | -0.02 |
| A1 |  |  | -0.04 |  |  | 0.07 | 0.17 | 0.02 |  | 0.08 |  | 0.01 |  |  |  | 0.01 |
| V1 |  | 0.99 |  |  |  | 0.9 | 1.19 |  | 1.73 | 2.2 |  |  | 1.12 | 0.59 |  |  |
| nonM1 |  |  |  |  |  |  | -8.24 | -0.02 | 0.07 | 0.05 |  |  |  |  |  |  |
| MnonM1 |  |  |  |  |  |  |  |  |  |  |  |  | -1.16 |  |  |  |
| CD1 |  |  |  |  |  |  | -0.25 | -0.02 |  |  |  |  |  | -0.14 | -0.05 |  |
| A/V1 |  |  |  |  |  |  |  | 0.05 |  |  |  |  |  |  |  |  |
| F2 |  |  |  |  |  |  | -0.03 | -0.005 |  | 0.13 |  |  |  |  | -0.01 |  |
| I2 |  |  |  |  |  |  |  |  |  |  |  |  |  |  |  |  |
| A2 |  |  |  |  |  |  |  |  | 0.6 | 0.31 |  | 0.03 | -0.5 | -0.27 |  |  |
| V2 |  |  |  |  | 1.22 | 0.8 |  |  |  |  |  |  |  |  | -0.27 |  |
| nonM2 |  |  |  |  |  |  |  |  |  |  |  |  | -0.22 | -0.08 |  |  |
| MnonM2 |  |  |  |  |  |  |  |  | -13.12 |  |  |  |  |  |  |  |
| CD2 |  |  |  |  |  |  |  |  |  | -0.39 | -0.09 | -0.01 |  |  |  |  |
| A/V2 |  |  |  |  |  | 0.74 | 0.58 |  |  |  |  |  |  |  |  |  |
| F3 |  |  |  |  |  |  |  |  |  |  |  | -0.02 |  |  | 0.07 |  |
| I3 |  |  |  |  |  |  |  |  | -0.48 |  |  | -0.07 |  |  |  | 0.04 |
| A3 |  |  |  |  |  |  |  |  |  | -0.2 | -0.04 | -0.01 |  |  |  |  |
| V3 |  |  |  |  |  |  |  |  |  |  |  | 0.27 |  |  |  | 0.22 |
| nonM3 |  |  |  |  |  |  |  |  |  |  |  |  |  |  |  |  |
| MnonM3 |  |  |  |  |  |  |  |  | -1.26 | -0.6 |  |  |  |  |  |  |
| CD3 |  |  |  |  |  |  |  |  | 0.35 |  |  | 0.08 |  | -0.03 | -0.08 | -0.04 |
| A/V3 |  |  |  |  |  |  |  |  |  |  |  |  |  |  |  |  |
| F4 |  |  |  |  |  |  |  |  |  |  |  |  |  |  |  | 0.01 |
| I4 |  |  |  |  |  |  |  |  |  |  |  |  |  | -0.33 | -0.13 | 0.07 |
| A4 |  |  |  |  |  |  |  |  |  |  |  |  | 0.22 | 0.22 | 0.15 | 0.01 |
| V4 |  |  |  |  |  |  |  |  |  |  |  |  | 1.12 | 0.61 | 0.19 |  |
| nonM4 |  |  |  |  |  |  |  |  |  |  |  |  |  |  |  |  |
| MnonM4 |  |  |  |  |  |  |  |  |  |  |  |  | 6.35 | 4.61 | 1.59 |  |
| CD4 |  |  |  |  |  |  |  |  |  |  |  |  | -0.42 |  |  | -0.09 |
| A/V4 |  |  |  |  |  |  |  |  |  |  |  |  |  |  |  | 0.04 |

Note: F – Frequency; I – intensity; A – arbuscules; V – vesicles; nonM – nonmycorrhizal areas; MnonM – mycorrhizal/nonmycorrhizal areas report; CD – colonization degree; A/V – arbuscules/vesicles report. Numbers 1, 2, 3, 4 and 5 indicates the phenophase: B1 – phenophase of 2-4 formed leaves; B2 - phenophase of 6 formed leaves; B3 - phenophase of 8-10 formed leaves; B4- cob formation phenophase; B5 - phenophase corresponding to physiological maturity.

Supplementary Table 3. Stepwise regression models for forecasting the colonization parameters in treated plants

|  | F2 | I2 | A2 | V2 | F3 | I3 | A3 | V3 | F4 | I4 | A4 | V4 | F5 | I5 | A5 | V5 |
| --- | --- | --- | --- | --- | --- | --- | --- | --- | --- | --- | --- | --- | --- | --- | --- | --- |
| Intercept | 50.55 | 25.94 | 637.3 | 0.15 | 70.92 | 1735.42 | 2.31 | 0.25 | -16.17 | -13.04 | -0.42 | -0.62 | 36.1 | 28.21 | -6.03 | 0.9 |
| F1 |  |  |  | -0.001 | -0.1 | -0.07 |  |  | -0.12 | -0.09 |  |  |  | -0.04 |  | -0.007 |
| I1 |  |  | -6.35 |  |  | -16.67 | 0.07 |  |  |  |  | 0.008 |  |  |  |  |
| A1 | -0.17 | -0.11 |  | 0.003 | -0.12 | -0.23 |  | 0.003 |  | 0.15 | 0.09 |  |  |  |  |  |
| V1 | 1.86 | 1.64 | 1.85 |  |  |  | 0.53 | 0.03 |  |  |  | -0.18 | 2.12 | 1.62 |  |  |
| nonM1 |  |  | -6.35 |  |  | -17.06 |  |  | 0.62 | 0.4 |  |  |  |  |  |  |
| MnonM1 |  |  | 0.27 |  |  |  |  |  |  | -0.74 | -0.39 | 0.08 |  |  |  |  |
| CD1 | 0.09 | 0.05 |  |  | 0.32 |  |  |  | 0.85 | 0.54 | 0.05 |  |  |  | -0.04 | 0.005 |
| A/V1 |  |  | -0.17 |  |  |  |  |  | -0.85 | -0.6 | -0.23 |  |  |  | 0.22 |  |
| F2 |  |  |  |  |  |  |  |  |  |  |  |  |  |  |  |  |
| I2 |  |  |  |  | -0.19 |  |  | -0.003 | 0.26 |  |  | 0.01 | -0.23 | -0.14 | 0.06 |  |
| A2 |  |  |  |  |  | 0.1 |  |  |  |  |  | -0.1 |  |  |  |  |
| V2 |  |  |  |  |  |  |  |  |  | -0.38 | -0.26 | 0.04 |  |  | 0.24 |  |
| nonM2 |  |  |  |  |  |  | 0.93 |  |  |  | 1.09 | 0.66 |  |  |  |  |
| MnonM2 |  |  |  |  |  |  | 0.59 |  |  |  |  |  |  |  |  |  |
| CD2 |  |  |  |  | -4.29 |  |  |  | -287 | -6.2 | -2.28 |  |  |  | 1.56 |  |
| A/V2 |  |  |  |  | 0.56 |  |  |  |  | 0.44 | 0.16 | 0.07 | 0.54 | 0.33 | -0.11 |  |
| F3 |  |  |  |  |  |  |  |  |  |  |  |  |  |  |  |  |
| I3 |  |  |  |  |  |  |  |  |  |  |  |  |  |  |  |  |
| A3 |  |  |  |  |  |  |  |  |  |  |  |  |  | 0.08 | 0.07 |  |
| V3 |  |  |  |  |  |  |  |  | 0.76 | 0.22 |  | 0.08 |  |  | 0.3 |  |
| nonM3 |  |  |  |  |  |  |  |  |  |  | 0.16 | 0.05 | -0.46 | -0.25 |  |  |
| MnonM3 |  |  |  |  |  |  |  |  | -1.96 |  |  |  | -2.21 |  |  |  |
| CD3 |  |  |  |  |  |  |  |  |  |  |  |  |  |  |  |  |
| A/V3 |  |  |  |  |  |  |  |  | -4.38 | -2.45 | -0.79 | -0.41 |  |  |  |  |
| F4 |  |  |  |  |  |  |  |  |  |  |  |  | -0.13 | -0.17 | -0.35 | -0.003 |
| I4 |  |  |  |  |  |  |  |  |  |  |  |  |  |  |  |  |
| A4 |  |  |  |  |  |  |  |  |  |  |  |  |  |  |  |  |
| V4 |  |  |  |  |  |  |  |  |  |  |  |  | 0.13 | 0.08 | 0.07 | -0.01 |
| nonM4 |  |  |  |  |  |  |  |  |  |  |  |  |  |  |  |  |
| MnonM4 |  |  |  |  |  |  |  |  |  |  |  |  |  | 0.25 | 0.11 | -0.01 |
| CD4 |  |  |  |  |  |  |  |  |  |  |  |  |  | -0.37 | -0.33 |  |
| A/V4 |  |  |  |  |  |  |  |  |  |  |  |  | 0.2 |  |  |  |

Note: F – Frequency; I – intensity; A – arbuscules; V – vesicles; nonM – nonmycorrhizal areas; MnonM – mycorrhizal/nonmycorrhizal areas report; CD – colonization degree; A/V – arbuscules/vesicles report. Numbers 1, 2, 3, 4 and 5 indicates the phenophase: B1 – phenophase of 2-4 formed leaves; B2 - phenophase of 6 formed leaves; B3 - phenophase of 8-10 formed leaves; B4- cob formation phenophase; B5 - phenophase corresponding to physiological maturity.
